# Supplementary material for: Hepatitis C (HCV) therapy for HCV mono-infected and HIV-HCV co-infected individuals living in Nepal
Source: PLoS Negl Trop Dis. 2020 Dec 16;14(12):e0008931. doi: 10.1371/journal.pntd.0008931 (PMC7773414; doi:10.1371/journal.pntd.0008931)
Supplement: S1 Appendix — (DOCX) [file pntd.0008931.s001.docx]

**S1 Appendix. Phase 1, Interferon-based treatment**

See S1 Table

**INTRODUCTION**

Until 2014, there was no national program for surveillance, treatment, and prevention of HCV in Nepal. In May 2014, an Expert Consortium proposed to validate short-course interferon (IFN)-based HCV treatment for HCV monoinfected and HIV-HCV coinfected individuals in Nepal based on data suggesting favorable characteristics of the population for response to IFN-based therapy [1]. At that time, IFN and ribavirin were the only approved drugs in Nepal for HCV treatment and the DAAs remained out of reach. We developed guidance for immediate HCV treatment using shortened treatment with pegylated (PEG)-IFN and ribavirin when baseline HCV RNA level was low and when rapid virological response (RVR) could be achieved. With the support of Global Fund and Deutsche Gesellschaft für Internationale Zusammenarbeit (GIZ) GmbH (German Development Cooperation) and through Save the Children, we provided free access to HCV treatment for 600 patients.

**MATERIAL AND METHODS**

Patients

Patients were recruited from 6 opioid substitution therapy (OST) sites located in 4 cities: Biratnagar, Dharan, Pokhara, and Kathmandu, Nepal (Figure 1).

At outset, inclusion criteria were as follows: (i) qualifying for HCV treatment i.e. (a) HCV genotype 1 with detectable HCV viral load and confirmed fibroscan >8.5 or confirmed AST to platelet ratio index (APRI) > 0.7, (b) HCV genotype 3 with detectable HCV viral load with or without fibrosis; (ii) HIV-HCV co-infected patients fulfilling above criteria and with (a) undetectable HIV viral load, (b) CD4 %>14 (CD4 >200), (c) stable ARV regimen for minimum 8 weeks; (iii) Residing in either Kathmandu, Pokhara, Dharan or Biratnagar and willing and able to make weekly visits for care and research follow-up, as required for at least 1 year.

Exclusion criteria included: (i) patients unable to provide consent for inclusion in the study; (ii) pediatric patients (age < 18); (iii) current, active heavy alcohol intake; (iv) any social issues that precluded patients to maintain routine follow-up appointments; (v) meeting any relative or absolute contraindications for PEG-IFN/ribavirin therapy as determined by Investigators; (vi) antiretroviral (ARV) non-compliance or recent change in ARV without repeat viral load testing at least 8 weeks after change; (vii) severe anemia (Hb <8.0 for HCV mono-infected; <7.0 for HIV-HCV co-infected). These criteria were relaxed once DAAs became the treatment regimen, at which point exclusion criteria were limited to: i, ii, iv.

Treatment

During phase 1, initiated in May 2015, patients meeting all inclusion and non-inclusion criteria received PEG-IFN/ribavirin combination therapy. Length of therapy (12-24 weeks) was based on baseline predictors including HCV genotype and viral load, HIV status and liver fibrosis score, and RVR.

**RESULTS**

Through a rolling admission process, we identified only 46 patients who qualified for IFN-based therapy after the initial 500 (304 HCV mono infected and 196 HIV-HCV co-infected) were screened. Among non-eligible patients, 15% were HCV RNA negative, 34% had advanced liver fibrosis, 20% of HIV-infected had detectable HIV virus, 13% had genotype 1 with low fibrosis, and 13% had high HCV viral load. Median age of treated was 35 years [interquartile range (IQR) 32-40] and 91.3% were males (S1 Table). Genotype 3 was found in 71.7% of cases and genotype 1a was found in 26.1%. Median HCV viral load before treatment was 5.7 log IU/mL. Twelve patients (26.1%) were HIV-HCV coinfected, all had HIV viral load <44 copies/mL. Nineteen patients (41.3%) had compensated cirrhosis. All patients were treated using Peg-IFN/ribavirin, 34 (73.9%) for 12 weeks, all others for 24 weeks. Thirty-eight patients (82.6%) achieved SVR. Of 19 patients with compensated cirrhosis, 18 achieved SVR.

**DISCUSSION**

In phase 1, we showed that, as hypothesized, minimizing treatment duration (74% of patients were treated in 12 weeks) and with excellent tolerability, SVR (83%) was achievable with IFN-based regimens. By pivoting care off of existing OST sites where clients had access to peer-based support and HIV disease management tools, we succeeded despite social factors and active drug use. Restrictions on who could safely be treated with IFN-based therapy limited our treatment cohort just as DAAs came on the horizon out of India through collaborative agreements with Gilead. The stakeholders and investigators had a key opportunity to advocate for DAA access in Nepal and were uniquely situated to immediately incorporate DAAs into the protocol after minimal delay for ethics approval. We then expanded the target treatment group to all comers, regardless of fibrosis, HIV disease control and active drug and alcohol use. Here again, in Phase 2 and Phase 3 we achieved very high per protocol SVR rates of 99% and 97.4%, respectively, showing that even higher cure was achievable in presence of pejorative prognostic factors such as advanced liver disease (including decompensated cirrhosis) and HIV-HCV co infection with DAAs.

**REFERENCES**

1. Kinkel H-T, Karmacharya D, Shakya J, Manandhar S, Panthi S, Karmacharya P, et al. Prevalence of HIV, Hepatitis B and C Infections and an Assessment of HCV-Genotypes and Two IL28B SNPs among People Who Inject Drugs in Three Regions of Nepal. PLoS ONE. 2015;10: e0134455. doi:10.1371/journal.pone.0134455
