# Supplementary material for: Hepatitis C (HCV) therapy for HCV mono-infected and HIV-HCV co-infected individuals living in Nepal
Source: PLoS Negl Trop Dis. 2020 Dec 16;14(12):e0008931. doi: 10.1371/journal.pntd.0008931 (PMC7773414; doi:10.1371/journal.pntd.0008931)
Supplement: S1 Table — (PDF) [file pntd.0008931.s002.pdf]

S1 Table: Characteristics of patients treated by PEG-Interferon+ribavirin during phase 1 (n=46). Qualitative data are presented as numbers and percentages and quantitative variables as median and interquartile range [IQR].

**Demographics**		
Site		
	Biratnagar	-
	Dharan	10 (21.7)
	Kathmandu	25 (54.3)
	Pokhara	11 (23.9)
Male gender		42 (91.3)
Age, years		35 [32-40]
BMI		22.9 [22.3-25.1]
HIV-HCV coinfection		12 (26.1)
HBV coinfection		0
APRI score		0.80 [0.61-1.47]
Cirrhosis		19 (41.3)
HCV genotype		
	1a	12 (26.1)
	3	33 (71.7)
	Other	1 (2.2)
HCV viral load before treatment (log IU/mL)		5.7 [5.4-6.1]
**HCV treatment regimen and treatment response**		
HCV treatment regimen		
	PEG-Interferon+ribavirin	46 (100)
HCV treatment duration		
	12 weeks	34 (73.9)
	24 weeks	12 (26.1)
End of treatment response (ETR)		
	Deceased	-
	Detectable	3 (6.5)
	Drop-out	3 (6.5)
	Missed	4 (8.7)
	Out of contact	-
	Undetectable	36 (78.3)
Sustained virological response (SVR)		
	Deceased	-
	Detectable	5 (10.9)
	Drop-out	3 (6.5)
	Out of contact	-
	Undetectable	38 (82.6)
