# Supplementary material for: Hepatitis C (HCV) therapy for HCV mono-infected and HIV-HCV co-infected individuals living in Nepal
Source: PLoS Negl Trop Dis. 2020 Dec 16;14(12):e0008931. doi: 10.1371/journal.pntd.0008931 (PMC7773414; doi:10.1371/journal.pntd.0008931)
Supplement: Attachment — Submitted filename: Response to the reviewers_15-10-2020.docx [file pntd.0008931.s003.docx]

Response to the reviewers’ comments

Editor

*We would request that you remove the interferon-based analysis (phase 1) from the main article and abstract as this includes a small number of patients and we consider these data obsolete and unlikely to be applicable to future programmes. This is particularly important when considering generalisable lessons from the programme to other settings and in the consideration of factors associated with failure to achieve SVR. These data from phase 1 could instead be included in a supplementary appendix.*

**Response:**

We have made this change as suggested. Data from phase 1 are now presented in Supplementary Appendix (S1 appendix) and Supplementary Table 1 (S1 Table).

*We also ask that you include a focus on lessons for improving the care pathway since the (successful) management of HCV using DAAs is already established in several low and middle income settings.*

**Response:**

We appreciate this suggestion. We have more clearly detailed lessons learned that may be useful for improving the care pathway in the Discussion. We highlight the summary:

“Based on our experience, a Care Pathway for HCV care expansion in settings like ours may consider: OST-based care with a designated community focal (non-medical) leader and intermediate level provider with doctor oversight, broad serologic testing for HCV with reflex Hep C core Ag and APRI, Hepatitis B Ag and HIV testing, monthly visits (clinical with substance use and depression screening) with bimonthly support group and peer counseling by paid staff throughout care and until SVR with more intensive peer support for active substance users. Hep C core Ag testing at 12 weeks after completion for SVR rather than virologic testing would optimize outcomes analysis at rural sites. The reliability and durability of earlier SVR is an important area for research. Clinical care will be simplified with pangenotypic drug.”

*Finally figure 2 should be revised to improve readability.*

**Response:**

Please see modified Figure 2 with improved readability.

Reviewer #1

Major comments

*1. The use of rapid tests for identifying HCV or HIV carriers might be a concern in terms of sensitivity. The authors should discuss this issue.*

**Response:**

All screened individuals were tested with both HCV rapid test (J. Mitra HCV Tridot, reported sensitivity/specificity 100/100) as well as HCV RNA. There were 3 discordant tests among HIV infected individuals who had HCV rapid negative with HCV RNA detectable which is reported elsewhere. These patients were included in the study. By WHO recommendations, 2 HIV rapid tests were used – first Determine (100/98.9) followed by Unigold (sens/spec 99.8/99.9) to address this concern.

*2. A high rate of SVR was obtained even with the use of IFN/RBV, although the most abundant genotype is 1. On the other hand, it has been described that some G3b may harbor DAAs natural mutations, and this subtype circulates in the region. Is there any additional information of the genotype/subtype of HCV in the non-responders? The authors should also discuss the genotype/subtype distribution in the country. Even if the present HCV treatment is pangenotypic, some focal diversities might play a role in reducing the high rate of SVR.*

**Response:**

Investigators on this work published a prior study demonstrating predominantly HCV genotype 3 (59.8 %) along with HCV genotype 1 (40.2%) [6]. We have noted these details (line 106-107).

Our PCR assay (Sacace) was not able to differentiate GT 3b so this was not explored but we agree with this excellent point and have made note of this weakness of the study in our discussion:

“Subsequent to initiation of this study data has come to light suggesting that GT3b is associated with natural prevalence of NS5A polymorphisms. To date, GT3 subtype analysis has not been done in Nepal and was not feasible in our system which we recognize as a weakness in this study. Per-protocol data supports excellent virologic results. Patients who failed to achieve SVR and those lost to follow-up before SVR will benefit from Hep C core Ag testing with genotypic subtype and resistance testing for any with replicating virus.”

*3. No statistical comparison is performed between the SVR rates of the different phases, if the population groups are comparable.*

**Response:**

The comparison of the SVR rates between phase 2 and phase 3 is now presented (p<0.001). Moreover, we have performed univariable and multivariable regression analyses including comparison of phase 2 and 3. See Table 3 and comment in Results, Discussion:

“We performed univariable and multivariable analysis to explore factors associated with SVR at 12 weeks (Table 3). We found that treatment in the Kathmandu sites was associated with SVR compared to treatment at the two sites outside of the capitol (OR 3.12 [1.20-8.13; p<0.001].) Substance use was associated with failure to achieve SVR (0.21; [0.10-0.44; p<0.001]. There were more patients reporting substance use during protocol during Phase 3 (11%) than in Phase 2 (4%). Patients treated in Phase 3 were less likely to achieve SVR than those treated during Phase 2, (0.08 [0.02-0.23; p<0.001] a difference that remains after controlling for substance use. Demographics, liver disease measured by APRI, cirrhosis and HIV infection status and GT were not associated with SVR.”

“Due to delays, largely related to logistics surrounding the earthquake, the timeframe of our protocol was shifted. Phase 2 treatment was so delayed that Phase 3 encompassed a 3-times larger cohort (413 vs. 135). In addition this resulted in final SVR data collection extending beyond the funding mechanism. The volume and delays likely contributed to the high attrition before data collection during Phase 3.”

*Minor comments*

*4. The reference of the earthquake of 2015 in Figure 1 is somewhat confusing. It would be preferable to include a comment on the earthquake in the text in page 6 lines 105-107, and include in the figures only the geographic location of the centers and the center.*

**Response:**

We have modified Figure 1 as suggested and added commentary on the earthquake to the text.

*5. Page 6: line 111: the inclusion criteria should include HCV positivity by rapid test and a reference to Figure 2.*

**Response:**

These changes have been made. Of note, those patients with negative HCV rapid test but detectable HCV RNA were also included.

*6. Page 7 line 143: instead of HCV testing, it is HCV RNA detection and genotyping.*

**Response:**

HCV rapid assay was also done in addition to HCV RNA detection for all. This has now been added.

*7. Page 6: the discussion of the cost of DAAs should be revised in light of availability of generic drugs.*

**Response:**

We have noted this advance though maintain that the cost remains prohibitive for the average Nepali. The poverty line in Nepal is listed at USD 225 per year.

*Reviewer #2:*

*I think you should focus on care pathway. In my point of view, the authors should show what is the best way to get the highest SVR12. Frequency of visits ? why did you choose a visit every 2 weeks and not every week.*

**Response:**

We appreciate this suggestion and have added commentary in our discussion.

*How do you explain the SVR12 difference between phase II and phase III ?*

Response:

“We explored why Kathmandu treatment was protective and treatment in phase 3 vs. 2 and substance use was associated with failure…More patients outside of the capitol were involved in political campaigning during Phase 3 around the April 2016 elections which required travel out of the region so they failed to follow-up. In addition, during Phase 3 the government of Nepal introduced a campaign mid protocol to actively incarcerate people with pending legal cases from the distant past who were arrested and remanded because of their previous cases related to drug use and other petty crimes. Only 1 incarcerated patient was able to remain in the study after extensive work on behalf of the investigators which highlights HCV treatment in jail as a need area in Nepal, where the rate of drug users in prison populations is >10% and as high as 28% [12]. Substance use was higher during phase 3 and independently associated with dropping out of the protocol.”

*Can you do a multivariate analysis to predict the SVR12. The number of patients included is high and the number of variables to study is roughly ten to twelve : genotype, viral load, cirrhosis, alcohol, drug injection during the treatment, centre, phase, distance from the center, housing conditions, level of study, treatment during the earthquaker period......*

**Response:**

We draw your attention to our multivariate regression analysis now shared in Table 3.

*How can you help other countries that want to reproduce your experience ?*

**Response:**

This is now discussed in the DISCUSSION section (lines 515-522). See also response to editor comment #2.

*This paper analyses the feasibility of the treatment of IVDU with hepatitis C in Nepal.*

*-It has been previously shown that the results of hepatitis C treatment in low income countries is as good as in high income countries (Bull World Health Organ. 2012 Jul 1;90(7):540-50. doi: 10.2471/BLT.11.097147.) -I then think that the paper should focus more on the care pathway than on SVR12 -We can easily imagine that the results will be as good with the DAAs.*

**Response:**

We have added discussion to address this query:

“Based on our experience, a Care Pathway for HCV care expansion in settings like ours may consider: OST-based care with a designated community focal leader and intermediate level provider with doctor oversight, broad serologic testing for HCV with reflex Hep C core Ag and APRI, Hepatitis B Ag and HIV testing, monthly visits (clinical with substance use and depression screening) with bimonthly support group and peer counseling by paid staff throughout care and until SVR with more intensive peer support for active substance users. Hep C core Ag testing at 12 weeks after completion for SVR rather than virologic testing would optimize outcomes analysis at rural sites. The reliability and durability of earlier SVR is an important area for research. Clinical care will be simplified with pangenotypic drug.”

*-Then the per-protocol results are not very useful.*

**Response:**

Please see Table 3 with multivariable analysis associated with ITT SVR and discussion of factors associated with failed SVR by ITT.

We have discussed these limitations, highlighted here:

“Due to delays, largely related to logistics surrounding the earthquake, the timeframe of our protocol was shifted. Phase 2 treatment was so delayed that phase 3 encompassed a 3-times larger cohort (413 vs. 135). In addition this resulted in final SVR data collection extending beyond the funding mechanism. The volume and delays likely contributed to the high attrition before data collection during phase 3.”

*-We want to know what are the risk factors that reduce intention to treat SVR12 -How many people have worked on the project ? How many people have been hired (nurses....) ?*

**Response:**

Please see our multivariable analysis for ITT SVR in Table 3, now added. This was a comprehensive effort however the key team was already in place through OST sites and Nepali clinical teams. We made the linkages and provided salary support. Due to delays by the earthquake, we feel that our volume may have exceeded optimal capacity in phase 3 and also when the protocol ended, outstanding SVR data points were less likely to be obtained. We interpret this to favor paid staff specifically for HCV treatment and care retention.

We have added a comment summarizing our Personnel in the Methods section:

“In each city (4) we engaged 2 doctors (2 hepatologists overall and 6 general practitioners), 2 nurses, 1 community focal person, 1 community worker and 1 phlebotomist.”

*-I am not sure that the group 1 is very useful as nobody treat hepatitis C with this treatment nowadays.*

**Response:**

We have moved the data from Phase 1 to Supplementary Appendix (S1 Appendix) and Supplementary Table 1 (S1 Table).

*-Can you tell us when the different phases started and finished ? When the earthquaker and its consequences started and finished.*

**Response:**

Phase 2 (Treated March 2015-November 2016); a rolling enrollment largely interrupted by the earthquake resulting in a lower cohort during this phase.

Phase 3 (Treated August 2017-January 2019); 3x larger cohort to catch up after a smaller Phase 2 cohort.

The Gorkha earthquake was on April 25, 2015 with subsequent significant shocks in the following month and a slow return to normalcy into 2016.

The contract of our protocol was extended due to the earthquake until January 2019.

-The number of references in the bibliography section is very low. Is there another experience in another countries ? in IVDU ? what have we learned from the egyptian experience ?

**Response:**

We had done an exhaustive literature search at the initiation and during this protocol and there had not been published data on similar protocols applying DAAs in resource-poor settings at the outset in 2015. A repeated search now still shows a dearth of experience. However, we added some new references on experiences in resource-limited settings (Sultan et al, 2020; Min Thaung et al., 2020). We also stress and discuss the need for decentralized treatment scale-up (Cousien et al., 2017; Castro et al, 2020).

*-What can we expect from the pangenotypic treatment (to add in discussion section)?*

**Response:**

We appreciate this point and have added comments:

“Hep C core Ag testing at 12 weeks after completion for SVR rather than virologic testing will simplify outcomes analysis. As further data evolves validating the reliability and durability of earlier SVR, this should be taken into consideration. Clinical care will be simplified with pangenotypic drugs.”

Subsequent to initiation of this study data has shown that GT3 non-3a are associated with natural prevalence of NS5A polymorphisms. To date, GT3 subtype analysis has not been done in Nepal and was not feasible in our system which we recognize as a weakness. Eight of 10 patients with virologic failure and those lost to follow-up before SVR will benefit from genotypic subtype and resistance testing, after Hep C core Ag testing to identify replicating virus for the latter. Advocacy to obtain glicaprevir/pibrentasvir – not currently available in the region, may be important if GT non-3a is described and as DAA use is more prevalent.

*-80% of SVR12 in phase III in ITT analysis cannot be considered a good result.*

**Response:**

We draw your attention to the discussion around challenges faced during phase 3 that may have contributed to a greater loss to follow-up for SVR, analyzed as failure by ITT as answered above regarding comparison of phase 2 and 3.

*-There is no data explaining the difference between the phases II and 3 ? Is it due to the earthquaker ?*

**Response:**

Please see detailed answer above.
